# Serum Levels of BDNF in Patients with Adenoma and Colorectal Cancer

**DOI:** 10.1155/2021/8867368

**Published:** 2021-02-12

**Authors:** Zhe Wang, Shuang Wang, Yu Liu, Shan Gao, Yunqing Yu, Zhaolan Hu

**Affiliations:** ^1^Department of Anesthesiology, The Second Xiangya Hospital, Central South University, Changsha, China; ^2^Medical Research Center and Clinical Laboratory, Xiangya Hospital, Central South University, Changsha, China; ^3^Department of Anatomy and Neurobiology, School of Basic Medical Science, Central South University, Changsha, China

## Abstract

The present study is aimed at examining the serum levels of brain-derived neurotrophic factor (BDNF) and investigating its role in differential diagnosis of colorectal cancer (CRC). *Materials and Methods*. In a Chinese population, we conducted a case-control study to compare the diagnostic performance of serum levels of BDNF and carcinoembryonic antigen (CEA) for CRC. We enrolled 61 healthy controls, 31 patients with adenomas, and 81 patients with CRC. We explored the correlation between serum levels of BDNF and several pathological features, such as tumor differentiation and TNM staging. *Results*. The serum levels of BDNF were significantly (*p* < 0.0001) higher in patients with CRC (10.64 ± 3.84, *n* = 81) than in the healthy controls (4.69 ± 1.69 ng/mL, *n* = 61). Serum BDNF also correlated with tumor size, tumor differentiation, and TNM staging (*p* < 0.05). For early diagnosis, the combination of BDNF (AUC 0.719; 95% CI, 0.621–0.816) and CEA (AUC 0.733; 95% CI, 0.632–0.909) slightly improved the diagnostic performance for CRC (AUC 0.823; 95% CI, 0.737-0.909). *Conclusions*. Combined detection of serum BDNF and CEA may thus have the potential to become a new laboratory method for the early clinical diagnosis of CRC.

## 1. Introduction

Colorectal cancer (CRC), one of the most common malignancies in western countries, is a frequent cause of death, and its prevalence is increasing worldwide [1]. The progression of the disease is slow, and the prospect for a reduction in mortality by early detection is much better than for most other forms of cancer [2]. Clinical screening of CRC relies mainly on a guaiac-based fecal occult blood test (gFOBT), which is limited, and the need for stool sampling may restrict acceptance and compliance [3, 4]. Moreover, intensive efforts have been made to identify blood-based biomarkers that may provide a promising alternative for noninvasive CRC screening [5]. Carcinoembryonic antigen (CEA) is currently the most commonly used serum tumor marker for CRC [6]. However, it is not recommended as a screening or diagnostic tool for this neoplasm, especially in the early stages, because of low sensitivity [7]. Thus, it is important to search for new serum markers for colorectal cancer.

Brain-derived neurotrophic factor (BDNF), a member of the neurotrophin family, is known to play a critical role in the modulation of cell survival, differentiation, and apoptosis in the nervous system [8], and it is also widely expressed in nonneuronal tissues [9]. Besides its distribution in healthy tissue, BDNF has also been found in tumors, such as neuroblastoma [10], breast cancer [11], lung cancer [12], hepatocellular cancer [13], and others. Interestingly, the binding of BDNF to its receptor, tyrosine kinase receptor B (TrkB), promotes the proliferation, invasion, and metastasis of tumor cells and induces tumor immunosuppression [14, 15]. A study assessing the mRNA and protein levels of BDNF and TrkB reported significantly higher levels in neoplastic tissue compared to adjacent nonneoplastic tissue from the same individual [16], suggesting that BDNF may play an intricate role in CRC. Previous studies have reported that the BDNF/TrkB pathway enhances several key biological processes in CRC cell lines, including proliferation, migration, and epithelial-mesenchymal transition, as well as resistance to apoptosis [16–18].

Despite its emerging role in tumorigenesis, few reports mention BDNF as a potential diagnostic marker in CRC. Here we describe a retrospective study utilizing an ELISA-based immunoassay to measure the protein concentration of serum BDNF in adenomas and patients with CRC and to investigate its potential usefulness as a blood-based biomarker for the diagnosis of CRC.

## 2. Materials and Methods

### 2.1. Patients and Healthy Controls

The subjects were chosen from among patients with CRC and adenoma admitted into the Second Xiangya Hospital of Central South University, from November 2018 to November 2019. All disease diagnoses were verified by pathological and cytological methods. None of the subjects were receiving radiation chemotherapy and other immunotherapy. Tumor node metastasis (TNM) staging was based on the Criteria of Colorectal Cancer Staging from the 8th edition of the Cancer Staging Manual from the American Joint Committee on Cancer (AJCC) [19]. The healthy controls (HC) were selected randomly from the Second Xiangya Hospital of Central South University.

### 2.2. Processing of Serum Samples

Fasting blood work was collected from each patient on the morning of the second day after hospitalization. The serum was obtained by centrifugation at 1500 × g for 10 min at 4°C. 500 *μ*L of samples were stored per tube at -80°C. Similarly, serum samples were collected from healthy control subjects on the morning of a routine examination.

### 2.3. Clinical Parameters and Laboratory Results

The clinical parameters of eligible patients, including age, sex, tumor location, TNM classification, tumor grade, and treatment type were retrieved from the patient's medical records. CEA measured by electrochemiluminescence using an Elecsys 2010 (Roche, Basel, Switzerland) were also collected from the medical records. The serum level of mature BDNF was measured by an ELISA-based method, as described previously [20], using the human BDNF ELISA Kit (cat no. ab99978, Abcam, Cambridge, United Kingdom). The optical density of each well was measured using an automated microplate reader (Epoch BioTek Instruments, Winooski, USA).

### 2.4. Statistical Analysis

All statistical analyses were performed with SPSS software (SPSS version 22.0, IBM, USA). A *p* value of less than 0.05 was considered statistically significant. Continuous variables were presented as mean ± standard deviation and were compared using the Mann–Whitney test. Variables displaying nonnormal distribution were logarithmically transformed (natural logarithm) before use in parametric analyses. The correlations between the serum levels of BDNF and CEA and clinicopathological tumor parameters, such as infiltration, metastasis, and differentiation, were analyzed using Pearson's correlation coefficient, chi-squared test, or Fisher's exact test. Specificity and sensitivity of serum BDNF and CEA expression levels for CRC patients with control of follow-up were evaluated with receiver operating characteristic (ROC) curve analysis. The diagnostic accuracy of biomarkers was also determined by obtaining the largest possible area under the curve (AUC) in ROC analysis. Bonferroni data were analyzed using SPSS v.23.0 software. For all comparisons, *p* < 0 05 was considered statistically significant. Graphs were plotted using GraphPad Prism v.6.0 software (GraphPad Inc., La Jolla, CA, USA).

## 3. Results

### 3.1. Clinical Characteristics of Study Participants

A total of 173 serum samples were collected, including 81 patients with CRC, 31 patients with adenomas, and 61 HC. The characteristics of the participants are presented in Table 1. The mean age in the patient cohort was 54 years (IQR 47-63 years). The gender distribution was 60.4% males and 39.5% females. Since the measurement of serum BDNF is influenced by the total serum protein concentration and the number of platelets [21], we measured these serum parameters in the patients and the HC and found no differences between the two groups. We also observed no significant difference in gender and age between the patient cohort and the HC.

The histological type of CRC and adenomas were defined according to the pathological diagnosis. TNM staging was based on the eighth edition of the TNM staging system (8-TNM) [19]. Patients did not receive chemotherapy or radiation before admission. The category and staging of TNM in patients with CRC are shown in Table 2.

### 3.2. Serum Level of BDNF in Patients with CRC

**Figure 1:**
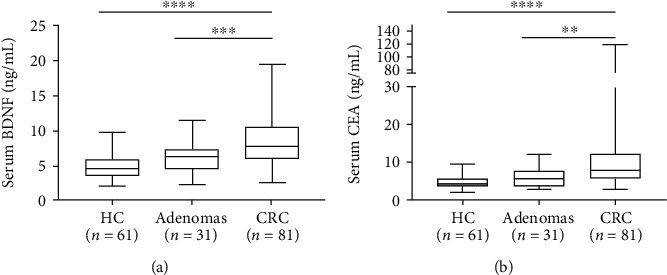
(a) Serum levels of BDNF and (b) serum levels of CEA in patients with colorectal cancer (CRC) as compared with the healthy controls (HC) and patients with adenomas. Serum levels of BDNF (ng/mL) in HC (*n* = 61), patients with adenomas (*n* = 31), and patients with CRC (*n* = 81). Asterisks indicate values that are significantly different compared to that in the healthy group (^∗∗∗∗^*p* < 0.0001; ^∗∗∗^*p* < 0.001).

**Figure 2:**
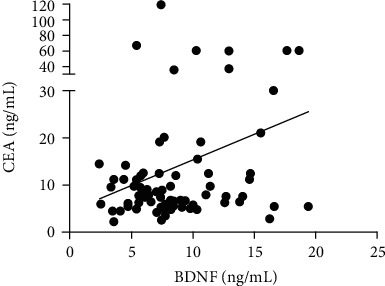
The corrections between BDNF and CEA in patients with CRC. There was a positive correlation between the serum BDNF and the serum CEA (*r* = 0.2304; *p* < 0.05).

Analysis of the serum levels of BDNF revealed that the serum level of BDNF in patients with CRC (10.64 ± 3.84 ng/mL) was significantly higher than the level in HC (4.69 ± 1.69 ng/mL; *p* < 0.0001) and patients with adenomas (5.97 ± 2.09 ng/mL; *p* < 0.001) (Figure 1(a)). Moreover, the mean serum level of CEA in patients with CRC was significantly higher (15.08 ± 19.11 ng/mL) than the level in HC (2.52 ± 0.42 ng/mL; *p* < 0.0001) and patients with adenomas (6.11 ± 2.43 ng/mL; *p* < 0.005) (Figure 1(b)). Moreover, we identified a slightly significant association between the serum level of BDNF and other biomarkers, such as CEA (*r* = 0.2304; *p* = 0.0385) in patients with CRC (Figure 2).

### 3.3. Correlation between Serum Level of BDNF or CEA and the Clinicopathological Parameters of CRC

The correlations between serum levels of BDNF or CEA and clinicopathologic features are summarized in Table 3. There was no significant correlation between BDNF (*p* = 0.489) or CEA (*p* = 0.848) expression and tumor location. However, high serum levels of BDNF (*p* = 0.036) were significantly correlated with tumor differentiation (Table 3). Serum levels of BDNF (*p* = 0.017) were also significantly correlated with tumor masses (Table 3), while CEA (*p* = 0.193) levels were not (Table 3). Serum levels of BDNF were significantly higher in patients with CRC with metastasis (6.78 ± 3.68 ng/mL) compared to patients without metastasis (10.30 ± 5.76 ng/mL) (*p* = 0.007, Table 3), suggesting that BDNF was related to tumor progression and metastasis in CRC.

### 3.4. Assessment of Tumor and Adenoma by ROC Analysis

**Figure 3:**
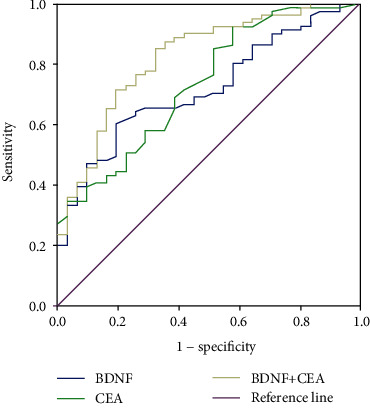
Receiver operating characteristic (ROC) curves comparing serum levels of BDNF, CEA, and a combination of BDNF and CEA in patients with adenoma vs. CRC.

To evaluate the potential of using serum levels of BDNF as CRC biomarkers, we analyzed the ROC curve and area under the curve (AUC). The cutoff values for BDNF and CEA in patients with recrudescent CRC were 7.36 ng/mL and 4.59 ng/mL, respectively (Figure 3). The sensitivity and specificity of these cutoff values were 92.6% and 4.19% for CEA (AUC 0.733; 95% CI, 0.632–0.909), respectively, and 60.5% and 80.6% for BDNF (AUC 0.719; 95% CI, 0.621–0.816), respectively. Combining CEA and BDNF, the sensitivity and specificity reached 85.2% and 67.7% (AUC 0.823; 95% CI, 0.737-0.909), respectively (Table 4). These results suggest that designating BDNF as an additional biomarker might be useful for the early detection of CRC. In particular, combining BDNF with CEA may enhance the sensitivity and specificity of CRC diagnoses.

## 4. Discussion

The early screening and diagnosis of CRC can help guide clinical measures for targeted treatment, as colorectal cancer progresses slowly and has a long incubation period. Over 90% of colorectal cancers originate from adenomas (a precancerous lesion), and it takes 5-7 years to transition from adenoma to cancer [22]. Thus, early detection that distinguishes the occurrence of colorectal adenoma is an important guarantee for the survival of patients. At present, research has not found that it is 100% sensitive and specific as tumor biomarkers. So, the combination of multiple biomarkers to diagnose tumors is a clinical trend in the future.

BDNF is the most abundant neurotrophin in brain tissue, and the serum level of BDNF has been suggested to function as a biomarker in some psychiatric and neurological diseases [23–25]. BDNF has also been found in a wide range of healthy nonneuronal tissues in adult humans [9]. For example, Shibayama and Koizumi [9] reported the existence of BDNF in the small intestine and colon.

However, high levels of BDNF are thought to be associated with more aggressive malignant behavior and a poor prognosis in human cancer [26]. Additionally, local progression, nodal and distant metastases, clinical stage, and poor prognosis are associated with increased TrkB levels, the high-affinity receptor of BDNF [27]. It has been shown that suppression of the BDNF/TrkB pathway could overcome resistance to therapy [28]. Moreover, reports indicate that BDNF is abnormally expressed in human CRC cells [18, 29]. Although serum levels of BDNF have been considered to have diagnostic value in several human cancers, there is a deficiency in its application for the diagnosis of CRC.

We found that the levels of BDNF in the serum of CRC patients were higher than those of HC, which was different from Brierley et al.'s research [30]. An explanation for this is that tumor staging and metastasis may affect the serum BDNF level. It has been shown that the level of BDNF is positively correlated with tumor metastasis and poor prognosis [27], and binding of BDNF to its receptor TrkB can promote the proliferation, invasion, and metastasis of tumors [14, 15, 27]. In addition, sample size, race, and individual differences may lead to different results.

Furthermore, we evaluated the performance of BDNF for the diagnosis of CRC and explored the relationship between BDNF and different clinicopathologic features. Our analysis revealed that the serum levels of BDNF were significantly higher in patients with CRC compared with those of HC, which were consistent with the upregulation of its expression in CRC tumor tissues. Our analysis also showed that the serum levels of BDNF performed better than CEA in distinguishing patients with CRC from patients with nonmalignant disease. We further analyzed the correlations between serum BDNF and clinicopathologic features of CRC and found that BDNF overexpression was closely associated with tumor differentiation, tumor size, and metastasis, but not with gender or age.

ROC analysis revealed that when CRC is compared with adenomas, the serum levels of BDNF provided a diagnostic efficacy with an AUC of 0.719, indicating that serum BDNF may likely have a potential role in the early diagnosis of CRC. The combination of the levels of BDNF and CEA could compensate for the deficiency of a single biomarker, and could increase the specificity and sensitivity for the diagnosis of CRC. Additionally, with different clinicopathologic features, such as differentiation and T stage, the diagnostic performance of BDNF was still better than CEA.

There are some limitations to this study. First, we are aware that the sample size in this cohort is rather small, which limits the power of multivariate analyses. Additionally, we failed to exclude patients with cirrhosis, which may interfere with the experimental results [31]. Therefore, further validation by larger-scale prospective trials is needed. Second, this study is a retrospective study and a prospective study is needed to examine the changes in serum levels of BDNF in high-risk populations, such as those with a family history of CRC, and the changes in stool properties to assess the applicability of BDNF in CRC monitoring.

## 5. Conclusion

Elevated serum levels of BDNF may play a critical role in the development and progression of CRC. Serum BDNF may serve as a convenient diagnostic biomarker with high efficiency, both alone and in combination with the level of CEA, to improve early diagnosis of CRC.

## Figures and Tables

**Table 1:** Baseline characteristics of healthy controls and patients with adenomas or CRC.

	HC subjects (*n* = 61)	Adenomas subjects (*n* = 31)	CRC subjects (*n* = 81)	*P* value
Gender (male/female)	38/23	19/12	49/32	N.S.^b^
Age (years)	55.5; 49.5-62.5	46.9; 36-45.5	54; 47-63	N.S.^a^
Total serum protein (g/dL)	66.7; 62.23-73	65.1; 62.23-70.1	68.7; 64.45-73.05	N.S.^a^
Platelets (no.: 10^3^/*μ*L)	182.5; 153.5-201.8	189; 145-207.7	177; 130-209	N.S.^a^
Total serum BDNF (ng/mL)	4.70; 3.52-5.67	5.97; 4.45-7.205	8.75; 5.88-10.64	<0.05

Mean standard deviation. HC: healthy control; CRC: patients with colorectal cancer; NS: nonsignificant. ^a^The Mann–Whitney *U* test (shown as median; Q1-Q3). ^b^*χ*^2^ square (shown as mean ± standard deviation).

**Table 2:** Baseline characteristics of colorectal cancer cases according to 8-TNM.

	Total (*n*)	Gender		Age	
		Male (*n*)	Female (*n*)	<60 years	≥60 years
TNM category					
T					
T1	2	1	1	1	1
T2	7	4	3	5	2
T3	41	17	24	24	17
T4	31	20	11	8	23
N					
N0	18	11	7	10	8
N1	27	14	13	18	9
N2	36	24	12	23	13
M					
M0	39	23	16	22	17
M1	42	26	16	30	12
TNM staging					
I	3	1	2	2	1
II	11	7	4	7	4
III	25	15	10	12	13
IV	42	26	16	30	12

*Note*. T: refers to the depth of tumor cell infiltration; N: refers to the level of lymph node metastasis; M: refers the condition of metastasis.

**Table 3:** Correlation between serum levels of BDNF or CEA and clinicopathological features of patients with CRC.

Clinical features	BDNF mean ± SD (range) (ng/mL)	*P*	CEA mean ± SD (range) (ng/mL)	*P*
*Gender*		0.484		0.111
Male (*n* = 49)	8.79 ± 4.95		17.26 ± 27.24	
Female (*n* = 32)	8.33 ± 5.52		17.32 ± 44.34	
*Age*		0.052		0.421
<60 (*n* = 51)	9.33 ± 5.19		16.45 ± 28.91	
≥60 (*n* = 30)	7.37 ± 4.94		18.70 ± 43.42	
*Tumor location*		0.489		0.848
Colon (*n* = 48)	8.93 ± 5.19		20.66 ± 42.40	
Rectum (*n* = 33)	8.14 ± 5.15		12.38 ± 18.32	
*Differentiation*		0.036		0.969
Low (*n* = 16)	5.75 ± 2.53		6.41 ± 6.01	
Middle (*n* = 58)	9.22 ± 5.60		21.46 ± 40.22	
High (*n* = 7)	10.10 ± 3.33		7.58 ± 6.31	
*T stage*		0.017		0.193
T1-2 (*n* = 9)	7.77 ± 4.58		4.07 ± 5.20	
T3 (*n* = 41)	7.17 ± 4.48		20.56 ± 44.38	
T4 (*n* = 31)	10.75 ± 5.55		16.78 ± 22.77	
*N stage*		0.134		0.086
N0 (*n* = 18)	7.64 ± 3.79		3.98 ± 5.19	
N1 (*n* = 27)	7.44 ± 5.14		31.02 ± 55.36	
N2 (*n* = 36)	9.97 ± 5.54		12.89 ± 18.77	
*Metastasis*		0.007		0.001
M0 (*n* = 39)	6.78 ± 3.68		3.54 ± 4.20	
M1 (*n* = 42)	10.30 ± 5.76		30.05 ± 44.63	
*TNM stage*		0.024		0.001
I-II (*n* = 13)	7.00 ± 3.76		1.86 ± 1.26	
III (*n* = 27)	6.65 ± 3.71		4.48 ± 4.95	
IV (*n* = 30)	10.31 ± 5.76		30.05 ± 44.63	

**Table 4:** Diagnostic efficiency of the biomarkers to differentiate between healthy controls, adenomas, and CRC.

Test variable	Sensitivity (%)	Specificity (%)	Youden index
CEA	92.6	41.9	0.35
BDNF	60.5	80.6	0.41
BDNF + CEA	85.2	67.7	0.53

## Data Availability

The data used to support the findings of this study are available from the corresponding author upon request.
